# Supplementary material for: Non-Volatile Terpenoids and Lipophilic Flavonoids from Achillea erba-rotta Subsp. moschata (Wulfen) I. Richardson
Source: Plants (Basel). 2023 Jan 15;12(2):402. doi: 10.3390/plants12020402 (PMC9866263; doi:10.3390/plants12020402)
Supplement: Supplementary file 1 [file plants-12-00402-s001.zip › plants-2094085-supplementary.pdf]

Supporting information

Non-Volatile Terpenoids and Lipophilic Flavonoids from *Achillea erba-rotta* Subsp. *moschata* (Wulfen) I. Richardson

Stefano Salamone [1,2], Nicola Aiello [3], Pietro Fusani [3], Antonella Rosa [4], Mariella Nieddu [4], Giovanni Appendino [1,*] and Federica Pollastro [1,2,*]

[1] Department of Pharmaceutical Sciences, University of Eastern Piedmont, Largo Guido Donegani 2/3 28100 Novara, Italy
[2] PlantaChem Srls, via Amico Canobio 4/6, 28100 Novara, Italy
[3] Council for Agricultural Research and Economics, Research Centre for Forestry and Wood, Piazza Nicolini 6, 38123 Trento, Italy
[4] Department of Biomedical Sciences, University of Cagliari, Cittadella Universitaria, SS 554, Km 4.5, 09042 Monserrato, Italy
* Correspondence: giovanni.appendino@uniupo.it (G.A.); federica.pollastro@uniupo.it (F.P.)

Table of Contents

Figure S1: ^{1}H NMR (400 MHz) of xanthomicrol **1** in $CDCl_3$

Figure S2: ^{1}H NMR (400 MHz) of taraxasterol **2** in $CDCl_3$

Figure S3: ^{1}H NMR (400 MHz) of matricarin **3** in $CDCl_3$

Figure S4: ^{1}H NMR (400 MHz) of tanetin **4** in $CDCl_3$

Figure S5: ^{1}H NMR (400 MHz) of penduletin **5** in $CDCl_3$

Figure S6: ^{1}H NMR (400 MHz) of 1(10)-secocariophyllane **6** in $CDCl_3$

Figure S7: ^{1}H NMR (400 MHz) of apigenin **7** in CD_3OD

Figure S8: ^{1}H NMR (400 MHz) of canin **8** in C_3D_6O

Figure S9: ^{1}H NMR (400 MHz) of 1α,2β-epoxy-3β,4α,10 α-trihydroxyguaian 6α,12-olide **9** in $CDCl_3$

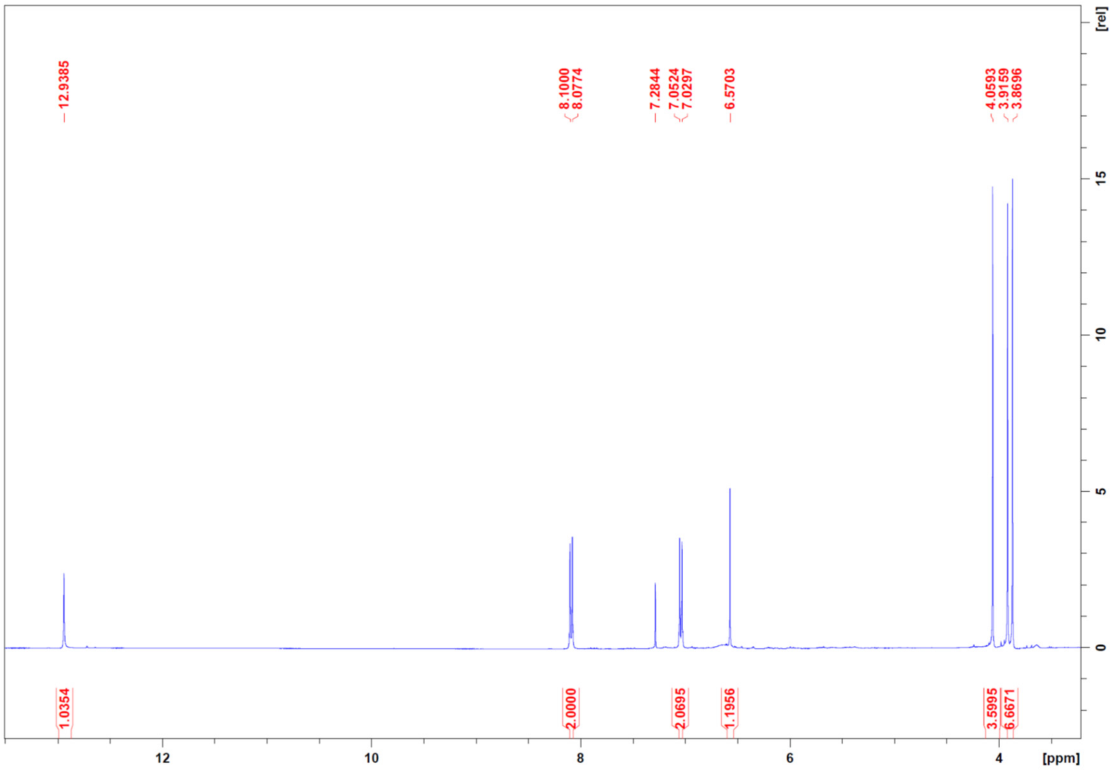

Figure S1: ^{1}H NMR (400 MHz) of xanthomicrol **1** in $CDCl_3$

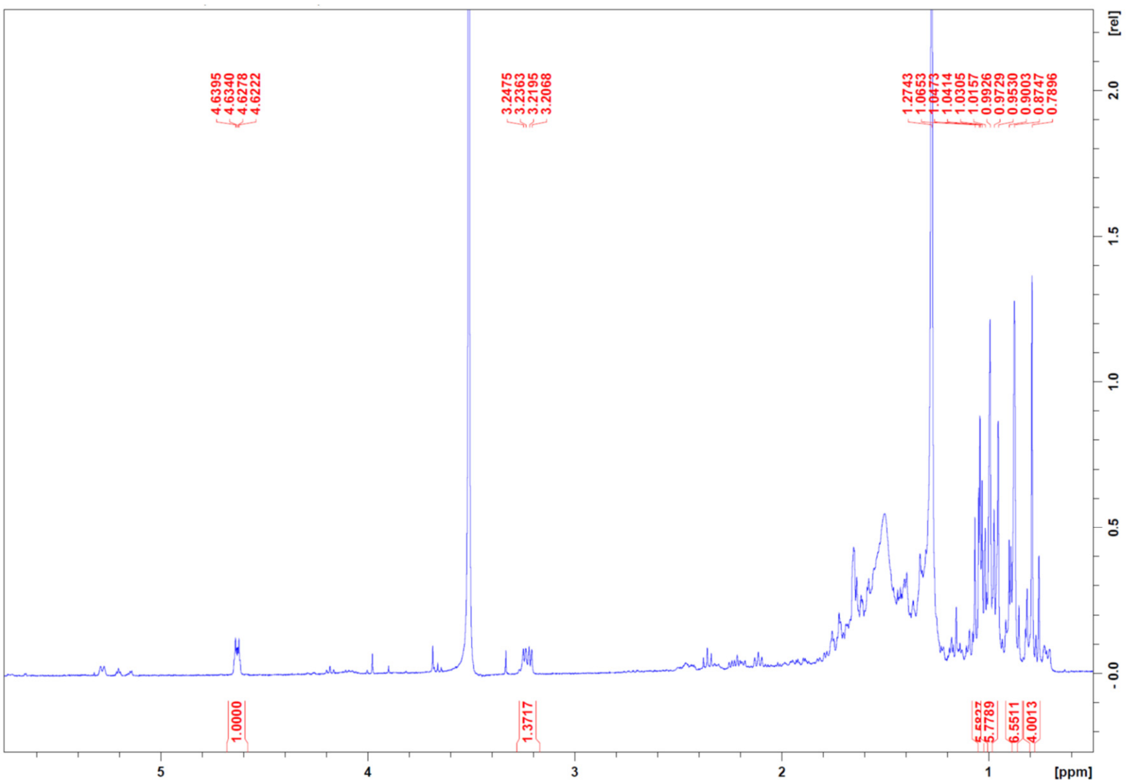

Figure S2: ^{1}H NMR (400 MHz) of taraxasterol **2** in $CDCl_3$

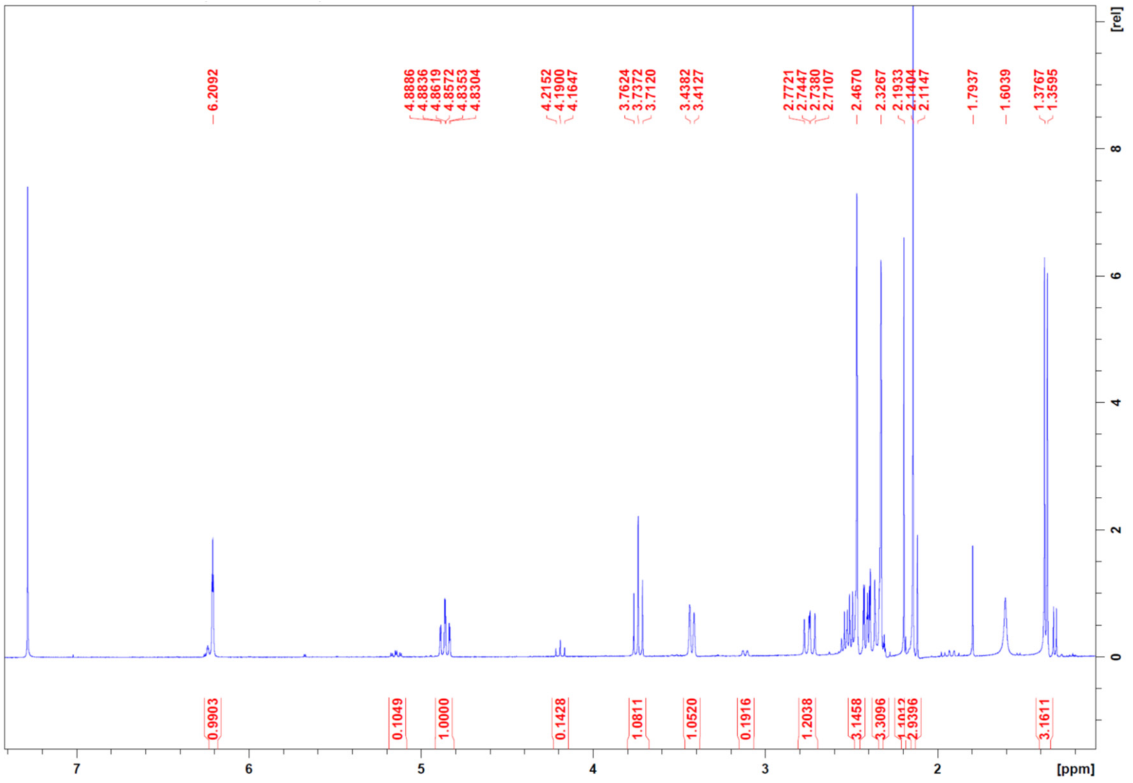

Figure S3: ^{1}H NMR (400 MHz) of matricarin **3** in $CDCl_3$

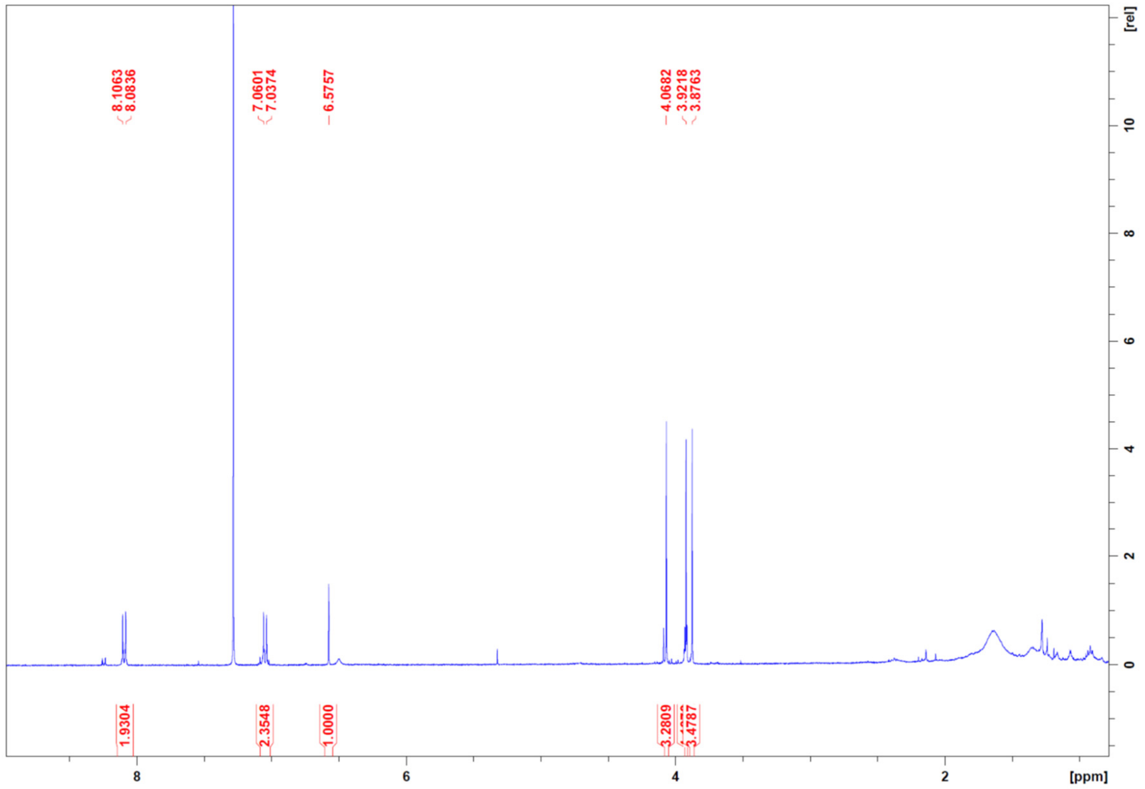

Figure S4: ^{1}H NMR (400 MHz) of tanetin **4** in $CDCl_3$

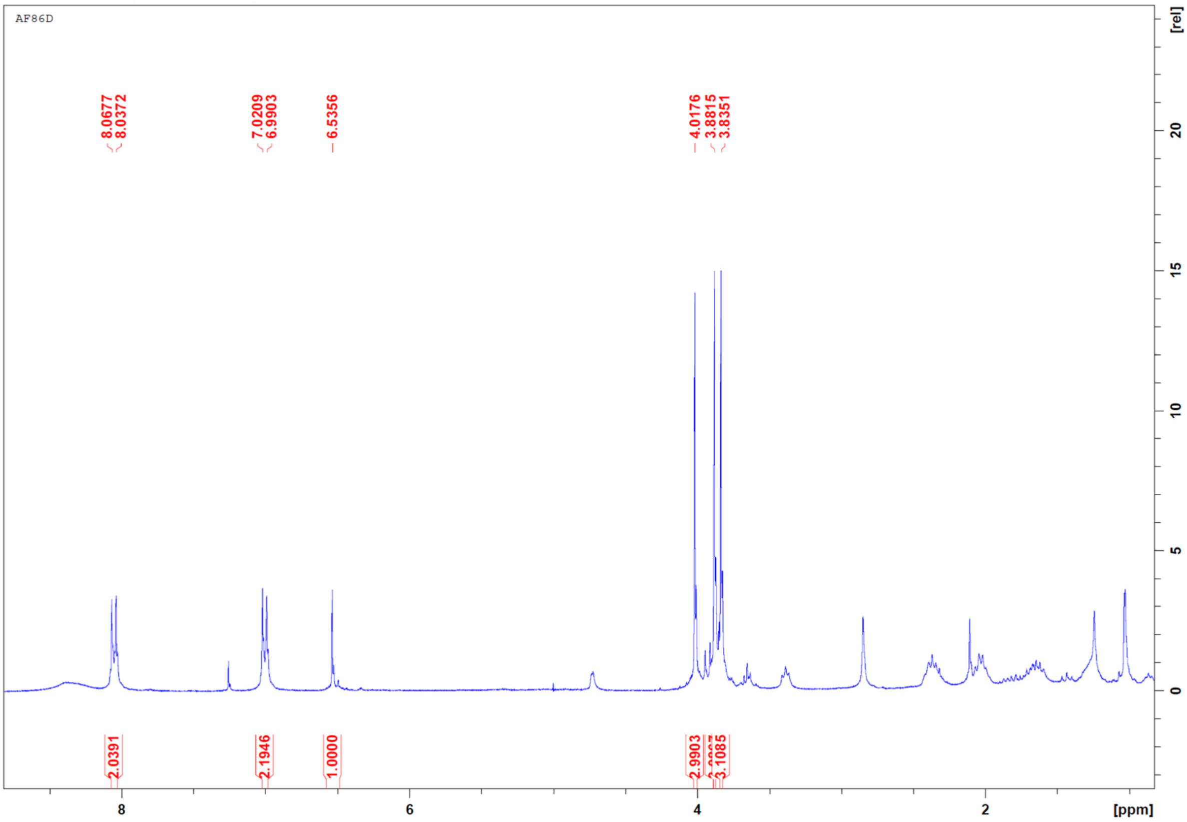

Figure S5: ^{1}H NMR (400 MHz) of penduletin **5** in $CDCl_3$

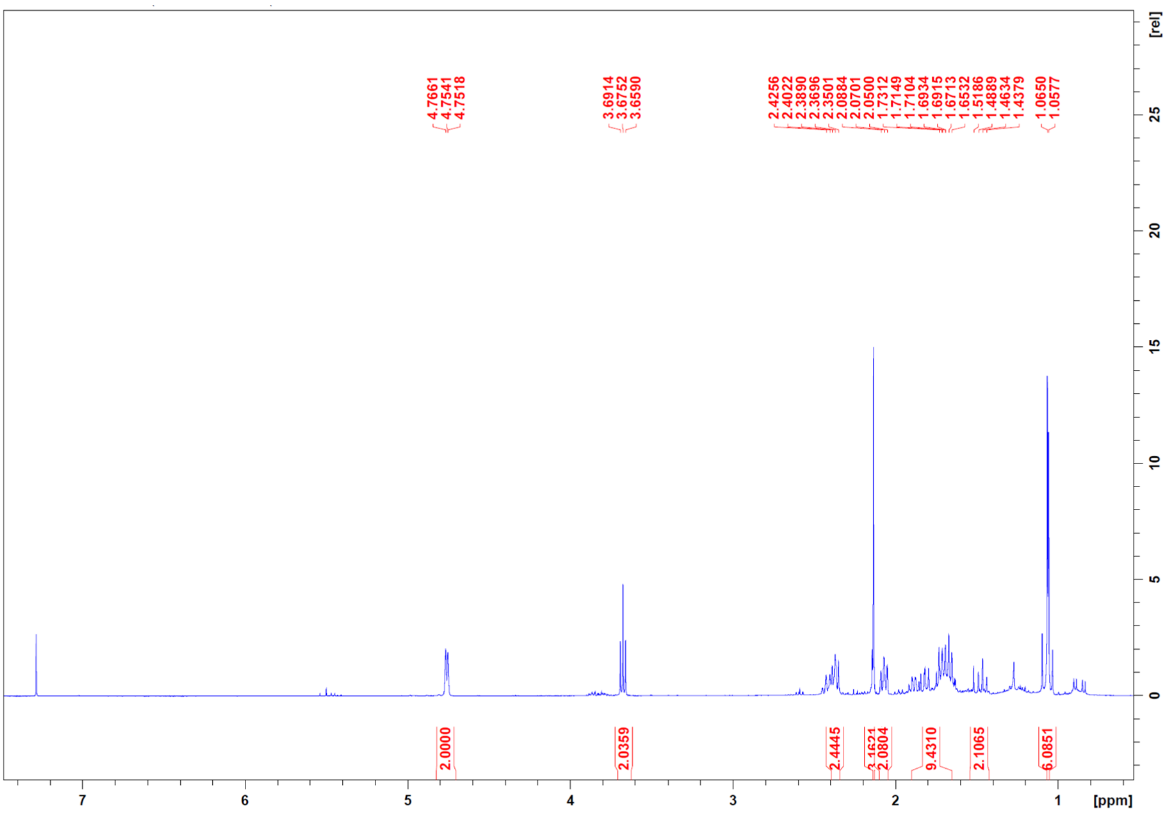

Figure S6: ^{1}H NMR (400 MHz) of 1(10)-secocariophyllane **6** in $CDCl_3$

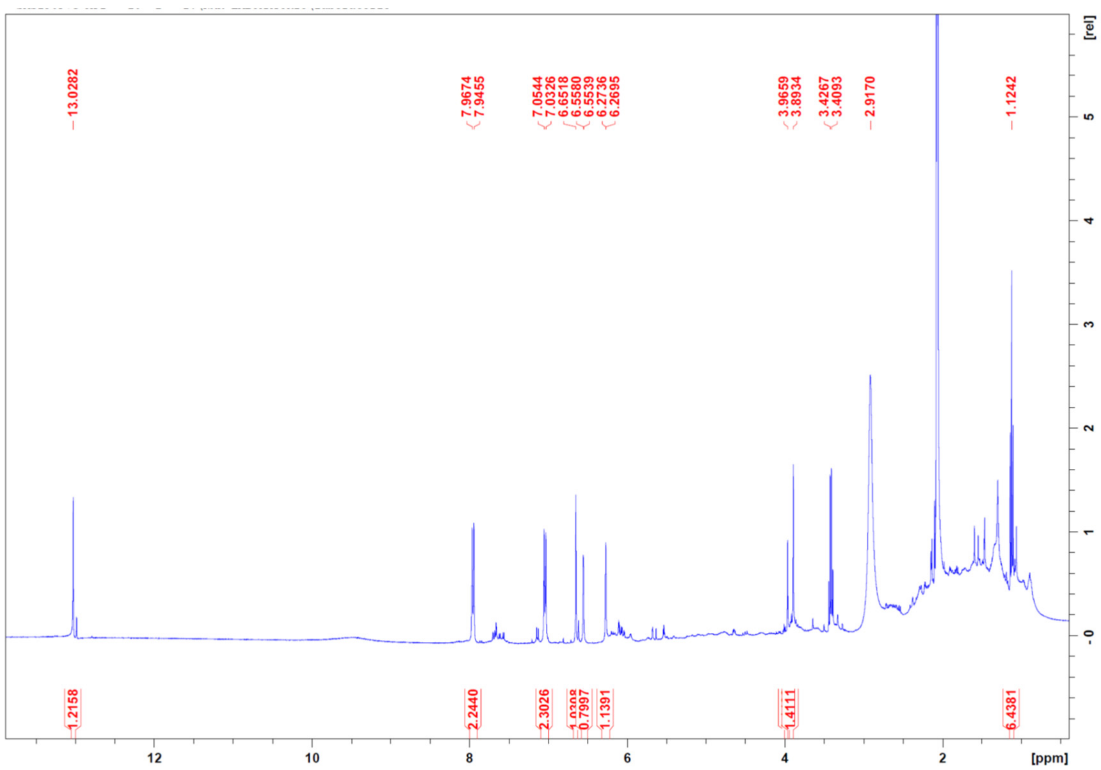

Figure S7: ^{1}H NMR (400 MHz) of apigenin **7** in CD_3OD

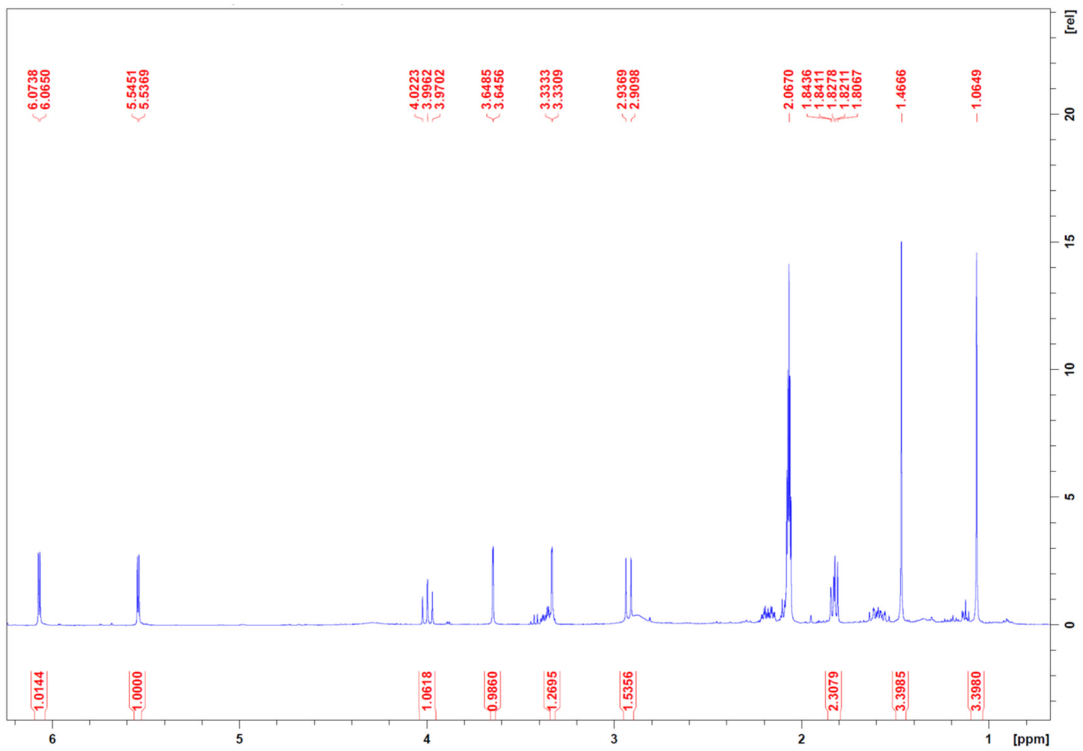

Figure S8: ^{1}H NMR (400 MHz) of canin **8** in C_3D_6O

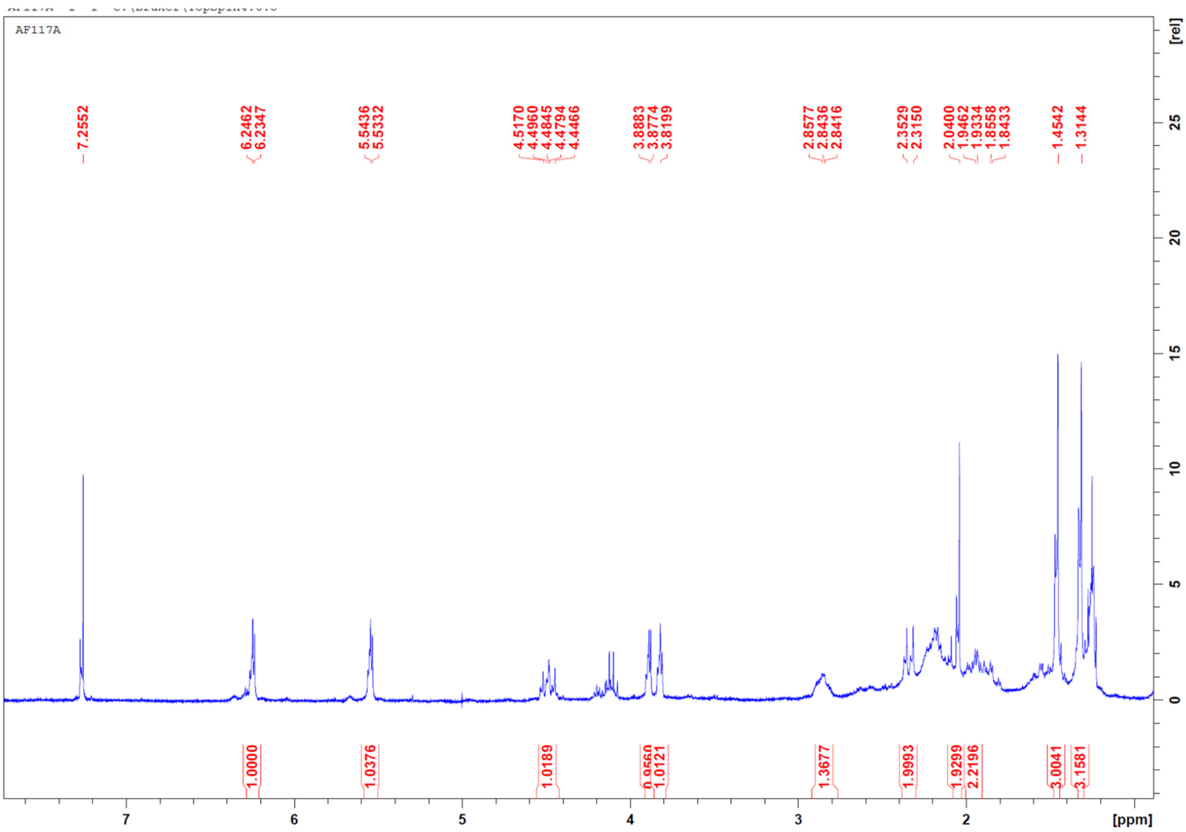

Figure S9: ^{1}H NMR (400 MHz) of 1α,2β-epoxy-3β,4α,10 α-trihydroxyguaian 6α,12-olide **9** in $CDCl_3$
